# GPS Tracking of Free-Roaming Cats (*Felis catus*) on SARS-CoV-2-Infected Mink Farms in Utah

**DOI:** 10.3390/v14102131

**Published:** 2022-09-27

**Authors:** Brian R. Amman, Caitlin M. Cossaboom, Natalie M. Wendling, R. Reid Harvey, Hannah Rettler, Dean Taylor, Markus H. Kainulainen, Ausaf Ahmad, Paige Bunkley, Claire Godino, Suxiang Tong, Yan Li, Anna Uehara, Anna Kelleher, Jing Zhang, Brian Lynch, Casey Barton Behravesh, Jonathan S. Towner

**Affiliations:** 1 Centers for Disease Control and Prevention, 1600 Clifton Road Ne, Atlanta, GA 30329, USA; 2 National Institute for Occupational Safety and Health, 1095 Willowdale Road, Morgantown, WV 26505, USA; 3 Utah Department of Health, 288 North 1460 West, Salt Lake City, UT 84114, USA; 4 Utah Department of Agriculture and Food, 4315 South 2700 West #4, Taylorsville, UT 84129, USA

**Keywords:** SARS-CoV-2, COVID-19, mink, *Neogale vison*, cat, *Felis catus*, GPS, mink farming, zoonosis, surveillance, spillover

## Abstract

Zoonotic transmission of SARS-CoV-2 from infected humans to other animals has been documented around the world, most notably in mink farming operations in Europe and the United States. Outbreaks of SARS-CoV-2 on Utah mink farms began in late July 2020 and resulted in high mink mortality. An investigation of these outbreaks revealed active and past SARS-CoV-2 infections in free-roaming and in feral cats living on or near several mink farms. Cats were captured using live traps, were sampled, fitted with GPS collars, and released on the farms. GPS tracking of these cats show they made frequent visits to mink sheds, moved freely around the affected farms, and visited surrounding residential properties and neighborhoods on multiple occasions, making them potential low risk vectors of additional SARS-CoV-2 spread in local communities.

## 1. Introduction

Coronaviruses are enveloped positive-sense RNA viruses belonging to the order *Nidovirales*, family *Coronaviridae* [1]. They are a diverse group of viruses capable of infecting several species of birds and mammals, including humans [2,3,4,5]. Severe acute respiratory syndrome coronavirus (SARS-CoV) emerged in human populations beginning in 2002 with the first known case of SARS identified in China [6,7]. Middle East respiratory syndrome coronavirus (MERS-CoV) was identified as the causative agent in severe respiratory infections similar to SARS-CoV in Saudi Arabia 10 years later [8]. A novel pathogenic coronavirus, severe acute respiratory syndrome coronavirus 2 (SARS-CoV-2), was identified as the causative agent after an outbreak of a severe human respiratory infection designated as novel coronavirus disease-19 (COVID-19) occurred in Wuhan, China in December 2019 [9]. This extremely transmissible virus quickly spread across China and into other countries and was declared a global pandemic by the World Health Organization (WHO) in March 2020 (see https://www.who.int/director-general/speeches/detail/who-director-general-s-opening-remarks-at-the-media-briefing-on-covid-19, accessed on 11 March 2020).

The first case of COVID-19 was identified in the United States in January 2020 [10], and as of August 2022 cases exceeded 95 million, with over 1,000,000 deaths (Centers for Disease Control and Prevention: https://covid.cdc.gov/covid-data-tracker/#trends_dailycases, accessed on 1 August 2022). In addition to primary concerns over human infections and control of transmission are the concerns about zoonotic SARS-CoV-2 spread from people to domestic animals and wildlife [11,12,13]. A variety of wild and some domestic animals have been reported as being potentially or definitively susceptible to infection with SARS-CoV-2, including white-tailed deer (*Odocoileus virginianus*), deer mice (*Peromyscus maniculatus*), horses (one seroconversion), pigs (mildly), and numerous others [3,14,15,16,17,18]. A wide variety of livestock (cattle, sheep, goat, alpaca, rabbit, chickens, and ducks), appear to be not susceptible to infection with SARS-CoV-2 [11,14,19,20]. However, American mink [Mustelidae, *Neogale* (*Neovison*) *vison* [21] farmed in Europe and the United States have shown to be highly susceptible to infection. European farms in the Netherlands, Denmark, and several other countries reported increased respiratory illnesses and mortality rates (2.4% in Netherlands, 0.45% in Denmark) among mink following human-to-mink transmission of SARS-CoV-2 [22,23,24]. Mink farms in the United States experienced similar outbreaks with mortality rates reaching as high as 55% of mink on affected mink farms in Utah [25]. In Europe and the United States, it was determined that the primary route of infection was human to mink. There was, however, documented spillover of human-acquired mink SARS-CoV-2 infection to humans on farms in Europe that led to the culling of all mink in Denmark [23,26].

Environmental sampling of infected mink farms and subsequent testing of those samples identified other potential transmission events involving infected mink and feral or free-roaming cats (Felidae, *Felis catus*) [22,24]. Cats have ACE2 receptors that are 85.39% identical to that in humans, suggestive of SARS-CoV-2 susceptibility [27]. Feline susceptibility to SARS-CoV-2 infection was initially detected among ill cats in households where owners had also become ill with COVID-19 [20,28,29,30]. Experimental infection of various domestic animals with SARS-CoV-2 confirmed that cats were highly susceptible to infection, whereas dogs and pigs showed lower susceptibility and could still become infected but not transmit SARS-CoV-2 to other animals [17,19,20,31]. Cat-to-cat transmission of the virus has been documented in experimental infection studies [19,31]. Recently, a probable cat-to-human transmission of SARS-CoV-2 occurred in Thailand when an infected cat sneezed on a veterinarian during an examination [32].

In August 2020, Utah state officials invited the Centers for Disease Control and Prevention (CDC) to collaborate on comprehensive onsite investigations of SARS-CoV-2 outbreaks among people and animals on two affected mink farms in Utah. Domesticated cats, both free-roaming and feral, are often present on or near mink farms [22,24,25], a practice frequently promoted by farmers as a form of rodent control. Based on the susceptibility of cats to infection with SARS-CoV-2 infection and their ability to transmit the virus to other cats, the August investigations included the sampling of 16 free-roaming or feral cats captured on or near one of the two affected farms. A total of eight cats tested positive for SARS-CoV-2: 1/16 (6.3%) by qRT-PCR, 4/16 (25%) by antibody reactive with SARS-CoV-2, and 3/16 (18.9%) by both PCR and reactive antibody (unpublished data [33]). These findings prompted a follow up investigation in October 2020 of affected farms, including the original farms and several newly affected farms, to assess the potential for infected cats to spread SARS-CoV-2 off-farm, including into surrounding communities. During the October investigations, cats were captured, and specimens were collected, including blood and oropharyngeal, nasal, body, and rectal swabs. Additionally, modified global positioning system (GPS) collars were attached to 15 cats and their movements were tracked for approximately one week. The results of this October 2020 sampling and GPS tracking are presented herein.

## 2. Materials and Methods

### 2.1. Animal Capture and Specimen Collection

Animal work was conducted under the approval of the CDC’s Institutional Animal Care and Use Committee (protocol 3104BARMULX), with the permission of the farm owners, the US Fish and Wildlife Service, the Utah Division of Wildlife Resources, and the Utah Animal Services. All cats were humanely captured using Tomahawk live traps (36 L × 10 W × 12 H; Tomahawk, Hazelhurst, WI, USA) baited with either canned cat food or tuna fish. Traps were set around the mink farms including along fence lines of pastures, near barns and outbuildings within the mink farming operational boundaries, and in and around the mink sheds. The cats targeted for capture were both adults and juveniles (estimated by body size to be approximately one year ± two months in age) and considered either feral (wild, independent, and untrusting of humans) or free roaming (wary and untrusting of humans, but still dependent on humans for food [i.e., mink feed]) [34,35]. Kittens were not utilized for this study. Captured cats were checked with a passive integrated transponder reader for any veterinary applied microchip, which would indicate domestic ownership or prior ownership. Traps were set and left overnight or set and checked at up to 30-min intervals depending on the farm and the cat populations residing there.

Captured cats were anesthetized prior to specimen collection using 3–5 mg/kg ketamine and 0.005–0.02 mg/kg dexmedetomidine, which was injected intramuscularly. Isoflurane was used as an inhalant as needed to maintain sedation. While under sedation, non-destructive sampling consisting of blood collection of up to 3 mL from either the jugular or medial saphenous veins. Oropharyngeal, body (fur), rectal and nasal swabs were collected using either sterile wire handled polyester-tipped applicators (nasal; Puritan Medical Products, Guilford, ME, USA) or sterile plastic polyester-tipped applicators (Life Technologies, Grand Island, NY, USA) and then placed into 0.5 mL viral transport medium and flash frozen in liquid nitrogen for storage until transportation on dry ice could be arranged. The cats were monitored continuously throughout the procedure. Atipamezole (0.005–0.02 mg/kg) was used to reverse dexmedetomidine to hasten anesthetic recovery time.

**Figure 1:**
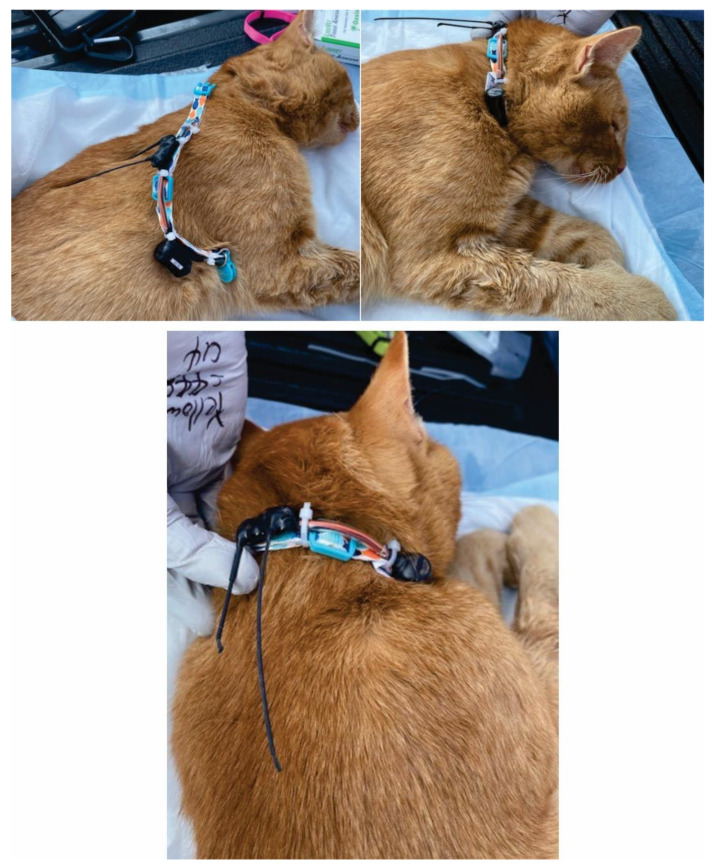
GPS collar placement on a free-roaming cat trapped on a mink farm in Utah in October 2020.

While the cats were still under anesthesia, a passive integrated transponder chip (PIT; Biomark, Boise, ID, USA) was inserted subdermally between the scapulae for future identification. The PIT tag was read using a handheld reader to ensure proper function and the number was recorded. The cat was then fitted with GPS data logger. The GPS logger (Telemetry Solutions, Concord, CA, USA) were manufactured for use with larger pteropodid bats but were modified to be attached to break away cat collars. The GPS loggers (<15.0 g) were attached to the collars and then the ensemble was adjusted for size depending on the size of the cat (Figure 1). After fitting the GPS collars, the anesthetized cats were placed in a shady area near the processing activities where they were allowed to fully awaken and wander off at their leisure under observation.

### 2.2. GPS Tracking

Base stations (Telemetry Solutions, Concord, CA, USA) capable of downloading GPS data wirelessly from the collars were set at strategic locations on the farms to allow for optimal line-of-site positioning on the farm. This typically involved mounting the base station on a light pole or long board to elevate the device. A 12 V lawnmower battery was used as an additional longer-term power source for the base stations. To conserve battery life in the GPS loggers (Telemetry Solutions, Concord, CA, USA), they were programmed to search for GPS positions at 20-min intervals for the first three days, 45-min intervals the fourth day, one-hour intervals for the fifth day, and then programmed to not take any positions to retain the remaining battery life for a wireless download should the logger come within the 2 km download range any time before the base stations were retrieved. An operational base station was placed on the dashboard of a vehicle on the day of retrieval to pick up and download any data from cats that did not return to the farm area after being collared but remained in the near vicinity (outside of the 2 km download range) of the farm. The base stations were collected between six and eight days after deployment depending on the farm and the deployment of the GPS collars.

### 2.3. GPS Data Analysis

**Figure 2:**
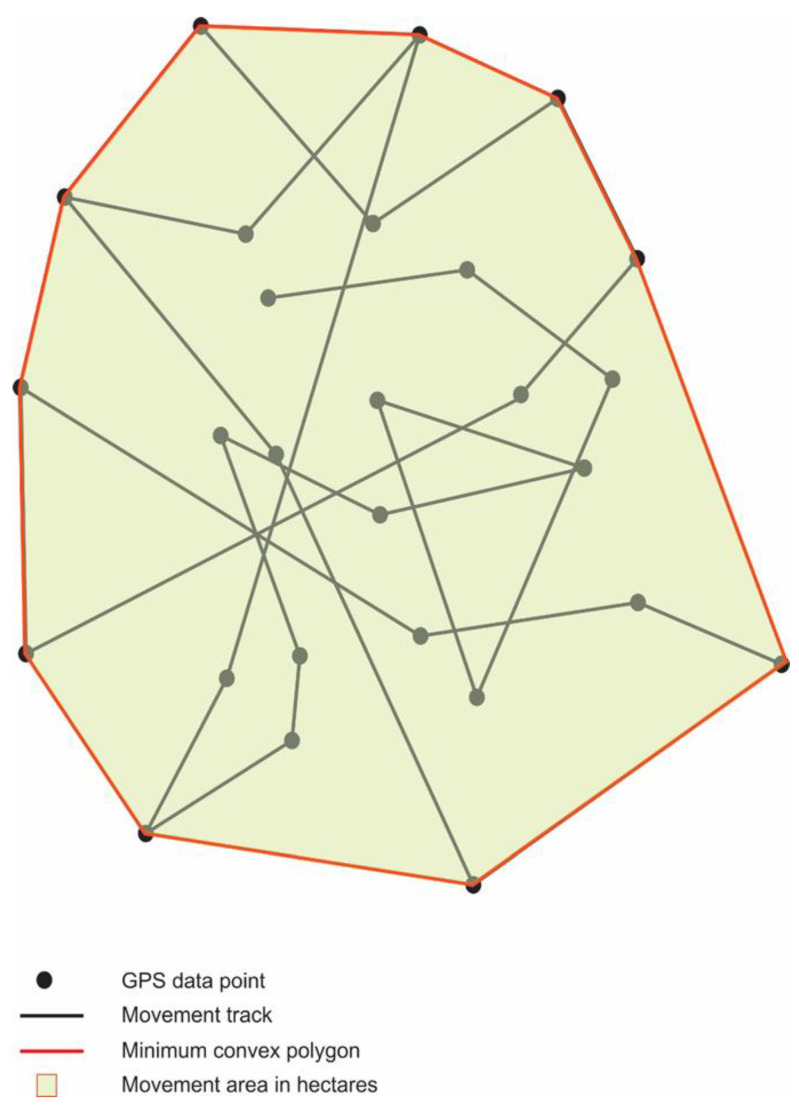
An illustration of a minimum convex polygon (MCP) encompassing the entire estimated potential movement area calculated from GPS data points and movement tracks This is an example of the type of polygon produced by the movement data of one GPS collared cat.

The GPS data were uploaded into a polygon generation program (Headwall Photonics, Bolton MA, USA; http://apps.headwallphotonics.com/, accessed on 21 February 2021) where minimum convex polygons (MCP; Figure 2) were created to encompass cat movements, and areas of movement were calculated using GPS data points. These polygon areas represent the minimum area in hectares (10,000 m^2^ = 1 ha) of cat movement (movement area) contained within the outermost GPS points collected by the GPS data logger. The data were also analyzed for activity within the convex hull polygon areas and characterized by mean values for number of visits to, and time (length of stay) in or near identifiable structures. Mink shed visits (MSV) are GPS data points within a mink shed. House visits (HV) are GPS data points within 30 m of a single house or up to three houses not considered a neighborhood. For this study, a neighborhood is defined as four or more houses in an area connected by a single, or multiple streets that are not designated as highways. Neighborhood visits (NV) are GPS data points and confirmed trajectories through a group of four or more houses in the area surrounding the mink farms. These GPS data points are further analyzed by time spent in the mink sheds, around homes, and in neighborhoods. The time spent in these areas is defined as the summation of the number of 20–60 min intervals a cat spent in that area as identified by the GPS coordinates and are characterized in the following manner: time in mink sheds (TMS) is the summation of time intervals identified in mink sheds, time at houses (TH) is the summation of time intervals identified within 30 m of a single house or up to three houses not considered a neighborhood, and time in neighborhoods (TN) is the summation of time intervals identified within a group of four or more houses in the area surrounding the mink farms. Movement and activity data were also analyzed according to SARS-CoV-2-infection status (based on PCR and antibody test results). All infection, activity, and movement data were analyzed using One Way Analysis of Variance (ANOVA) in SPSS Statistics v25 (IBM Corp, Armonk, NY, USA).

### 2.4. RNA Extraction and qRT-PCR

Nucleic acid extractions were performed with the Kingfisher Flex Instrument (ThermoFisher Inc., Waltham, MA, USA), using the MagMAX™ Viral/Pathogen Nucleic Acid Isolation Kit (Cat # A48310, ThermoFisher Inc., Waltham, MA, USA) according to the manufacturers’ instructions. The MVP_Flex_200ul protocol was used with an elution volume of 100 µL. Human specimen control (HSC; A549 cell suspension) was included as an extraction control and water as a negative control.

QRT-PCR testing of specimens targeting the N-gene of SARS-CoV-2 was performed on the ABI 7500 Fast Dx Real-time PCR system (Applied Biosystems, ThermoFisher Inc., Waltham, MA, USA) using the CDC influenza SARS-CoV-2 (FluSC2) multiplex assay (https://www.fda.gov/media/139743/download; kit Cat # Flu SC2-EUA, accessed on 29 April 2022). TaqPath™ 1-Step Multiplex Master Mix (No ROX) (Cat # A28522, ThermoFisher Scientific Inc., Waltham, MA, USA) was used to further test the qRT-PCR negative specimens for β-Actin to verify successful RNA extraction.

### 2.5. Serology

Blood specimens were tested for antibody reactive with SARS-CoV-2 using a mix-and-read assay following previously published protocols [36]. Briefly, serological reactivity against the receptor-binding domain of SARS-CoV-2 spike protein was determined using a species-independent mix-and-read assay (PMID: 34112850). Baseline reactivity in cats was established by analyzing adult cat serum samples (n = 108) collected prior to SARS-CoV-2 emergence in Texas, USA (PMID: 31972512). Based on those measurements, positive/negative cut-off value of 2.8 (fold over blank signal) was chosen (baseline set average signal 0.32, standard deviation 0.307, maximum 2.64).

### 2.6. Sequencing

In August 2020, only one qRT-PCR positive cat had sufficient vial load to produce whole genome SARS-CoV-2 nucleic acid sequence to compare to SARS-CoV-2-infected mink on the same farm. For this analysis, nucleic acid from 50 qRT-PCR positive specimens (49 mink and 1 cat) was subjected to Illumina MiSeq sequencing following previously published protocols [37]. Consensus sequences were generated using an iterative refinement meta-assembler IRMA algorithm [38].

### 2.7. Phylogenetic Analysis

Phylogenetic relations of the August 2020 investigation sequences were inferred using maximum likelihood analyses implemented in Treetime using the NextStrain pipeline [39]. Randomly selected Utah SARS-CoV-2 sequences from mink (n = 120), humans (n = 2701), and one cat with collection dates through 31 January 2021, and other Utah mink farm investigation-related sequences were downloaded from the GISAID public repository and used for comparison.

## 3. Results

### 3.1. Specimen Testing

In October 2020, 15 cats from three farms were PIT tagged, fitted with a breakaway GPS collar and released following specimen collection. Evidence of SARS-CoV2 infection was detected in a total of 8/15 (53.3%) cats sampled and fitted with GPS collars including 2/15 (13.3%) by PCR only, 4/14 (26.6%) by antibody reactive against SARS-CoV2, and 2/15 (13.3%) by both PCR and antibody (Table 1). Specimens positive by PCR included one oropharyngeal swab from an adult male and a body swab from each of three other cats: an adult male, adult female and a juvenile male. Antibody reactive against SARS-CoV2 was detected in 40.0% (6/15) of the cats sampled and fitted with GPS collars (Table 1). There were no significant differences between males and females or adults and juveniles with respect to SARS-CoV2 RNA positivity (age: F = 0.007, *p* > 0.9; sex: F = 0.149, *p* > 0.7) or antibody status (age: F = 0.200, *p* > 0.6; sex: F = 1.182, *p* > 0.2) among the cats sampled.

### 3.2. Sequencing and Phylogenetic Analysis

**Figure 3:**
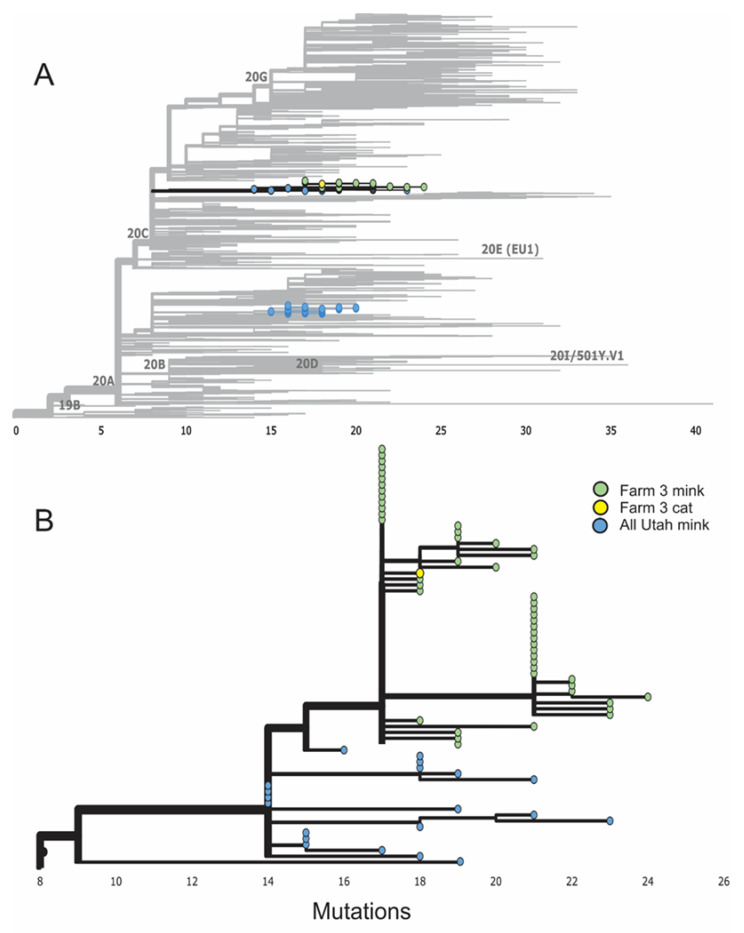
(**A**) Phylogenetic results of a maximum likelihood analysis showing 2822 randomly selected full genome SARS-CoV-2 sequences from mink (n = 120), humans (n = 2701), and one cat from Utah mink farms sampled in August 2020. (**B**) Exploded view of clade 20C containing sequences from Utah mink farms sampled in August 2020, showing nearly identical viral sequences obtained from cat and mink samples on one farm (farm 3).

Phylogenetic analysis of full genome SARS-CoV-2 sequences obtained in August 2020 from mink and cat samples on one farm clustered together when compared to other Utah mink and human SARS-CoV-2 circulating in the community at approximately the same time (Figure 3A). The single cat sequence from an oral swab sample was identical to some sequences obtained from mink on the same farm and differed from other mink sequences on the same farm up to seven single nucleotide polymorphisms (SNP; Figure 3B).

### 3.3. Activity and Movement

GPS data were recovered from 13 of the 15 cats collared for GPS tracking (Table 1). Analysis of the activity data (Table 2) identified significant differences between adults (n = 9) and juveniles (n = 4) where juveniles had higher mean values for the number of mink shed visits (MSV = 14.25, F = 8.493, *p* = 0.014) and overall time spent in mink sheds [TMS = 423.75 min (7.06 h)., F = 5.222, *p* = 0.043] than adults [MSV = 5.22, TMS = 48.89 min (0.81 h)]. Mean time spent in neighborhoods was also higher for juveniles (TN = 141.25 min) than adults (TN = 28.89 min), but the difference was not significant (F = 3.139, *p* = 0.104). Conversely, adults had larger mean values for home visits (HV = 47.0) and mean time spent near homes (TH = 2083.89 min) than juveniles (HV = 22.5 and TH = 762.50 min), but those differences were not significant (F = 2.330, *p* = 0.155 and F = 3.099, *p* = 0.106, respectively). The only activity variable between adults and juveniles that clearly did not show much difference was mean neighborhood visits (NV = 0.67 and NV = 1.25, respectively; F = 0.378, *p* = 0.546).

There were no significant differences detected in means of activities between males and females or between uninfected cats and those positive for SARS-CoV-2 RNA by PCR or that had antibody reactive to SARS-CoV-2. On average, cats with detectable SARS-CoV-2 RNA roamed 12.40 ha of area near mink farms where they spent 239.17 min in infected mink sheds during 8.0 visits, spent 1763.33 min at homes during 43.83 visits, and spent 33.33 min in neighborhoods over 0.67 visits. On average, cats with antibody reactive with SARS-CoV-2 roamed 12.72 ha of area near mink farms where they spent 286.00 min in infected mink sheds during 8.60 visits, spent 1580.00 min at homes during 34.20 visits, and spent 40.0 min in neighborhoods over 0.80 visits (Table 3).

## 4. Discussion

The 15 cats captured and GPS tagged in this study were located on three farms and considered free-roaming and not completely feral because of their tendencies to remain close to the mink farming operations, presumably to take opportunistic meals of mink feed in and around the mink sheds and to prey on rodents and other wildlife in and around the farming operations [34,35]. The cats were mostly tolerant of humans at a distance but were very wary and unapproachable. They would not be considered domesticated, although domestic household cats were present on some farms and could interact with the free-roaming cats. These potential interactions are important because both active infection and past infection with SARS-CoV-2 was detected in 8/15 (53.3%) cats around the mink farming operations. Observations and inquiries made to the farmers about the cats on their farms revealed that household pet cats did interact with the free-roaming cats.

Of the 15 cats captured and fitted with GPS collars, only 13 returned to the area or remained close enough to the base stations enabling wireless download of the data. The GPS loggers were attached to break away collars, so it is possible the two cats without data left the area and did not return, or the collars on these cats fell off or were otherwise removed. The GPS data collected from the other 13 cats revealed that infected cats frequently entered the mink sheds, moved through surrounding properties, and spent large amounts of time in multi-house neighborhoods where they could potentially encounter and have contact with other free-roaming cats, domestic pets, livestock, and peridomestic wildlife. One actively infected cat (C15) roamed over 50 ha while spending over 3 hours in neighborhoods while three other cats, all with detectable SARS-CoV-2 RNA on their fur, spent anywhere from 4–65 h visiting around homes. None of these three cats with detectable SARS-CoV-2 RNA on their fur were considered actively infected with SARS-CoV-2 and these findings represent environmental contamination only; there is no evidence that pet fur can serve as a fomite for SARS-CoV-2 transmission [40].

An age bias was detected with respect to activity by the cats in and around the mink farms. Juvenile cats were more likely to visit mink sheds and spend more time in the sheds. One possible explanation for this difference may be the readily available food source. Mink were fed a meat paste daily, which was deposited on top of the cage where younger cats would have easy access to an opportunistic meal. Research on social hierarchy and feeding in feral and free-roaming cats in Italy reported that juvenile cats were allowed to feed before the adults [41,42], which could explain the age biases detected in this study. The adult cats would let the juveniles feed first and at the same time the mink were feeding. There may have simply been less food available when the adults fed, resulting in fewer visits and less time spent in the mink sheds. Moreover, the mink feed as well as bedding in empty mink cages also attracted rodents and the juvenile cats may have found more success hunting in these sheds instead of competing with the adults outside of the sheds. Feral and free-roaming cats do exhibit territoriality, but in the presence of an abundant food supply, they tend to either lose this tendency or maintain smaller territories [43]. The latter may have been the case on the mink farms. It was evident that the older cats were proficient at rodent control at the farms where cats were present. Through personal observation and poor trap success during rodent trapping efforts, the rodent populations at these farms were greatly reduced compared to the to the farms that did not have cats present. The sparse rodent activity that was observed at the farms with cats present was predominantly inside the mink sheds. Despite the increased time in the mink sheds by the juveniles, significant differences in detectable SARS-CoV-2 RNA or antibody reactive with SARS-CoV-2 between adults and juveniles were not identified. This may be due to the small sample size or that the susceptibility of the cats to infection with SARS-CoV-2 means that even infrequent visits into the mink sheds by the adults lead to infection. Alternatively, cat to cat transmission independent of direct mink interactions may be the reason for finding no significant differences between adult and juvenile infections. There were also no significant differences detected in means of activities between uninfected and SARS-CoV-2-infected cats. This lack of statistically significant difference also may be due to the small sample size.

On average, juveniles spent more time in neighborhoods than did the adults and adults had more home visits and spent more time near homes, but neither of these measurements were statistically significant, perhaps again, due to the small sample size. Regardless of the significance with these age biases, the data clearly show that these infected free-roaming cats are dispersing from the SARS-CoV-2-infected mink farms, traveling as far as 1.8 km to nearby properties with homes and to multiple home neighborhoods, and spending large amounts of time there.

The phylogenetic analysis of the SARS-CoV-2 sequences from one infected cat oral swab sample and numerous mink samples on Utah farms revealed that the same virus was circulating in both species in August. The larger analysis (Figure 3A) did contain human sequences, including one from farm 3. While human-to-cat transmission of SARS-CoV-2 cannot be entirely ruled out, the fact that the sequence is from a free-roaming cat that was unapproachable by a human makes it very unlikely the cat was infected by the farmer, or any human, as close proximity or contact is needed for transmission of COVID. Many of the infections in these cats presumably occurred from their visitations inside the mink sheds where infections in the mink were extremely high [25,44]. However, experimental infections demonstrated that SARS-CoV-2-infected cats can transmit the virus to other cats [19,31]. This suggests that cat-to-cat transmission of SARS-CoV-2 outside of the mink sheds may have occurred, a finding further reinforced by the detection of SARS-CoV-2 on the fur of three free-roaming cats on the farms. SARS-CoV-2-infected cats on mink farms in the Netherlands were also thought to have been involved in spreading the virus to neighboring farms [20]. This transmissibility between free-roaming cats elucidates the potential risk of spreading infection to household pet cats, which could then enter a human dwelling.

Like humans, the potential of these free-roaming cats to shed SARS-CoV-2 and infect other animal species, including peridomestic and endangered wildlife, is also of concern [12,16]. Deer mice, a known peridomestic rodent species and a common prey species of both free-roaming and domesticated cats, are highly susceptible to SARS-CoV-2 infections and have been shown, experimentally, to not only become infected with SARS-CoV-2, but to shed and transmit the virus to naïve deer mice [14,15,45]. Moreover, other common peridomestic species of wildlife including striped skunks (*Mephitis mephitis*) and Bushy-tailed woodrats (*Neotoma cinerea*) were found to be susceptible to SARS-CoV-2 infection and shed virus in respiratory secretions [46].

An important concern from a One Health perspective is spillover into domestic animals or wildlife that could result in the establishment of a competent secondary zoonotic wildlife reservoir [13] or the emergence of mutations or variants of concern that could significantly impact disease severity, therapeutic effectiveness, or vaccine efficacy. While SARS-CoV-2 transmission from these peridomestic wildlife species to humans has not been documented, it is still a potential risk, albeit low, to public health for this zoonotic virus, where animal-to-human transmission has been documented with other animal species. Recently a white-tailed deer-to-human transmission of a SARS-CoV-2 variant was reported in Canada [47] and free-roaming cats can interact with deer in fields neighboring SARS-CoV-2-affected mink farms. The same is true for free-roaming and domestic cats. Their susceptibility to SARS-CoV-2 and capacity for shedding virus and transmitting it to naïve cats is of public health concern, especially considering the recent report of a probable cat-to-human transmission event [32] and the propensity of the SARS-CoV-2 virus to accumulate new mutations and then spill back into humans with potentially different transmission characteristics.

In summary, the results of this study demonstrate the susceptibility of free-roaming cats to infection with SARS-CoV-2 through their activities on infected mink farms. Furthermore, the results demonstrate the capacity of a SARS-CoV-2-infected cat to extend the potential risk of viral transmission to household pets, peri-domestic animals, wildlife, and into surrounding homes and multi-home neighborhoods over broad distances.

## Figures and Tables

**Table 1:** Results for SARS-CoV2 qRT-PCR and antibody testing for the 15 cats captured and fitted with GPS collars on mink farms.

Sex	Age	No. Collared	No. with GPS Data	No. PCR+	No. Ab+
Female	Adult	3	2	1	0
	Juvenile	2	2	0	1
Male	Adult	8	7	2	4
	Juvenile	2	2	1	1
Total		15	13	4	6

**Table 2:** Comparison of the mean activity and movement of adult (n = 9) and juvenile cats (n = 4) near SARS-CoV-2-infected mink farms.

Activity/Movement	Age	Mean	Standard Deviation	*p* Value
Mink shed visits (no.)	Adult	5.22	4.47	0.014
	Juvenile	14.25	6.65	
Home visits * (no.)	Adult	47.00	29.00	0.155
	Juvenile	22.50	19.33	
Neighborhood visits ** (no.)	Adult	0.67	1.41	0.546
	Juvenile	1.25	1.89	
Time in/near mink sheds (in minutes)	Adult	48.89	38.87	0.043
	Juvenile	423.75	518.83	
Time in/near homes (in minutes)	Adult	2083.89	1313.44	0.106
	Juvenile	762.50	1059.01	
Time in neighborhoods (in minutes)	Adult	28.89	67.16	0.104
	Juvenile	141.25	169.72	
MCP *** movement area (in hectares)	Adult	9.00	15.75	0.829
	Juvenile	7.19	5.91	

No. = number. * Home visits: visits to human inhabited dwellings such as houses, barns, outbuildings, etc. ** Neighborhood visits: visits to 4 or more houses in an area with non-highway surface streets. *** MCP= Minimum convex polygon containing outermost GPS points.

**Table 3:** Comparison of mean activity and movement with respect to SARS-CoV-2 infection status (measured by SARS-CoV-2 PCR and antibody tests) of cats (n = 13) near SARS-CoV-2-infected mink farms.

Activity/Movement	Infection Status	Mean	Minimum	Maximum	Standard Deviation	*p* Value
Mink Shed Visits (no.)	Ab−	7.63	2.00	15.00	4.17	0.087
	Ab+	8.60	0.00	24.00	9.91	
	PCR−	8.00	2.00	15.00	5.29	1.00
	PCR+	8.00	0.00	24.00	8.37	
Home visits * (no.)	Ab−	42.75	2.00	82.00	32.28	0.616
	Ab+	34.20	1.00	54.00	22.30	
	PCR−	35.71	2.00	82.00	29.76	0.626
	PCR+	43.83	1.00	79.00	28.22	
Neighborhood visits **	Ab−	0.88	0.00	4.00	1.46	0.935
	Ab+	0.80	0.00	4.00	1.79	
	PCR−	1.00	20.00	250.00	1.53	0.711
	PCR+	0.67	0.00	1195.00	1.63	
Time in/near mink sheds (in minutes)	Ab−	88.13	20.00	250.00	73.821	0.293
	Ab+	286.00	0.00	1195.00	512.39	
	PCR−	100.00	20.00	250.00	85.29	0.455
	PCR+	239.17	0.00	1195.00	469.59	
Time in/near homes (in minutes)	Ab−	1738.13	10.00	3915.00	1454.08	0.848
	Ab+	1580.00	5.00	2800.00	1334.48	
	PCR−	1603.57	10.00	3140.00	1384.28	0.843
	PCR+	1763.33	5.00	3915.00	1443.67	
Time in neighborhoods (in minutes)	Ab−	78.13	0.00	340.00	131.47	0.582
	Ab+	40.00	0.00	200.00	89.44	
	PCR−	89.29	0.00	340.00	137.85	0.403
	PCR+	33.33	0.00	200.00	81.65	
MCP *** movement area (in hectares)	Ab−	5.80	1.24	14.13	4.502	0.385
	Ab+	12.72	0.83	50.75	21.42	
	PCR−	5.23	0.83	14.13	5.25	0.366
	PCR+	12.24	2.27	50.75	18.97	

No. = number. * Home visits: visits to human inhabited dwellings such as houses, barns, outbuildings, etc. ** Neighborhood visits: visits to 4 or more houses in an area with non-highway surface streets. *** MCP = Minimum convex polygon containing outermost GPS points.

## Data Availability

The authors declare that all data supporting the findings of this study are available within the article and its supplementary information files or from the authors upon request.
